# Randomized cross-over trial of demand oxygen delivery system

**DOI:** 10.1097/MD.0000000000020010

**Published:** 2020-05-08

**Authors:** Tatsuya Nagano, Kazuyuki Kobayashi, Takashi Omori, Takehiro Otoshi, Kanoko Umezawa, Naoko Katsurada, Masatsugu Yamamoto, Motoko Tachihara, Yoshihiro Nishimura

**Affiliations:** a Department of Internal Medicine, Division of Respiratory Medicine; b Clinical and Translational Research Center, Kobe University Hospital, 7-5-2 Kusunoki-cho, Chuo-ku, Kobe, Hyogo, Japan.

**Keywords:** demand oxygen delivery system, long-term oxygen therapy

## Abstract

**Introduction:**

Long-term oxygen therapy is reported to improve hypoxemia and survival in patients with respiratory failure. The demand oxygen delivery system (DODS) saves oxygen and extends the usable time of an oxygen cylinder 2- to 3-fold. A portable oxygen concentrator with an auto-DODS has been developed to switch its sensitivity among 3 levels (standard, high, and extra high) and to supply pulsed-flow oxygen when it detects apnea. The aim of this study is to evaluate the efficacy of this newly developed portable oxygen concentrator with auto-DODS compared to the efficacy of conventional DODS in oxygenation.

**Methods and analysis:**

Twenty-six patients with chronic obstructive pulmonary disease or interstitial pneumonia will be randomized to use auto-DODS or conventional DODS at rest and during a 6-minute walk test. Primary endpoints are mean oxygen saturation (SpO_2_) at rest and during the 6-minute walk test. Secondary endpoints are the ratios of the times during which the oxygen concentrator operates at each sensitivity mode (standard, high, and extra high) and at a constant pulse rate to the examination time, the ratio of the times during which SpO_2_ fall below 90% to the examination time, the lowest value of SpO_2_ during the examination time, the mean and highest pulse rates during the examination time, 6-minute walking distance, recovery time, Borg scale, comfort, and reliability, which are measured by a numerical rating scale and a questionnaire, respectively.

**Ethics and dissemination:**

The study was conducted in accordance with the Declaration of Helsinki and was registered on Aug 23, 2019 (https://jrct.niph.go.jp/en-latest-detail/jRCTs052190041). The results of the study will be presented at academic conferences and submitted to a peer-reviewed journal.

**Trial registration number:**

jRCTs052190041

## 1 Introduction

### 1.1 Background and rationale

Long-term oxygen therapy (LTOT) is currently provided for patients with severe chronic respiratory failure, pulmonary hypertension, chronic heart failure, cyanotic congenital heart disease, and severe cluster headache. LTOT improves hypoxia and survival and is generally provided by an oxygen concentrator at home and a portable oxygen cylinder when going out. A respiratory synchronizer (demand oxygen delivery system, DODS) is a device that supplies oxygen from an oxygen cylinder only when the patient inhales; it saves oxygen and increases the usable time of the oxygen cylinder 2 to 3–fold. A previous study revealed that the SpO_2_ of the DODS when the patient is at rest was equivalent to that of a continuous flow system (93.7 ± 2.1% and 93.8 ± 1.9%, respectively) and that SpO_2_ of the high-flow DODS when the patient exerts effort to breathe was also equivalent to that of a continuous flow system (92.5 ± 2.8% and 93.1 ± 3.1%, respectively).^[1]^ In addition, the distance walked using oxygen with the DODS was equivalent to that from continuous flow system at 2 l/min (*P* = .72; CI 0.34–1.08).^[2]^ At present, DODS and continuous flow are generally regarded as having the same tendency regarding oxygenation at rest and on effort, and DODSs are widely used in general settings. Recently, a portable oxygen concentrator with an auto-DODS that can detect the negative pressure of inspiration by switching among 3 sensitivities (standard, high, or extra-high sensitivity) and that supplies oxygen only during inspiration has recently been developed to improve oxygenation and patient comfort. This oxygen concentrator can also supply pulsed flow for a fixed time when it detects apnoea. We hypothesize that this auto-DODS is superior to conventional-DODS in improving at rest and on effort desaturation.

### 1.2 Objectives

In this clinical trial, we will use a PULSOX Me 300 pulse oximeter with a built-in memory function to compare SpO_2_, pulse rate, walking distance, recovery time, exercise intensity, comfort and reliability between an auto-DODS and a conventional DODS, both equipped with a Hi-Sanso Portable α II portable oxygen concentrator, which detects the negative pressure of inspiration with standard sensitivity and sends an alert after 3 minutes without breathing, both at rest and on effort.

### 1.3 Trial design

This clinical trial is a single-institution, randomized, open-label, crossover, exploratory comparative study to examine the efficacy and safety of an auto-DODS in patients receiving LTOT.

## 2 Material and methods

### 2.1 Study setting

Twenty-six study subjects were randomly assigned to either an auto-DODS followed by a conventional-DODS or a conventional-DODS followed by an auto-DODS group (allocation ratio 1:1). The study period consists of 13 weeks, including a 4-week pre-observation period, 1 day of testing, a 4-week test interval, another day of testing and a 4-week post-observation period. During the study period, either the auto-DODS or the conventional-DODS was used only for 30 minutes at rest and for a 6 minute walk.

### 2.2 Eligibility criteria

The inclusion and exclusion criteria for the trial are listed in Table 1.

**Table 1:**
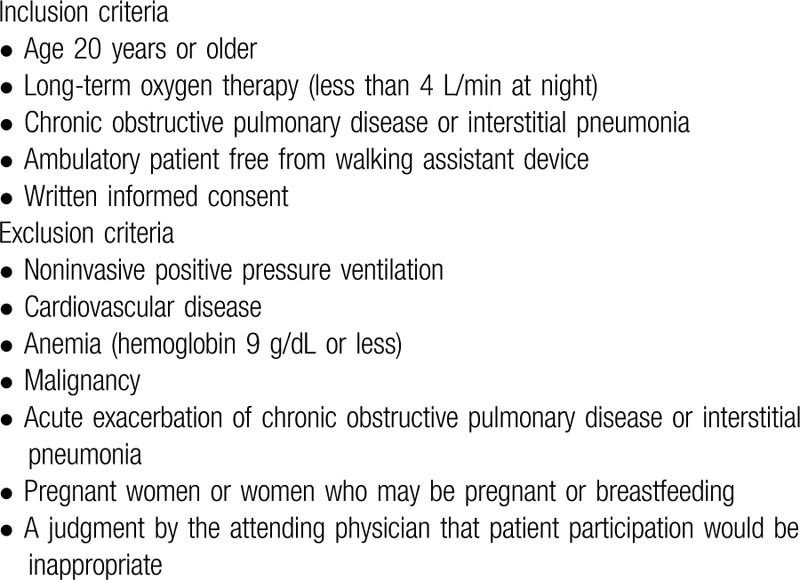
Inclusion and exclusion criteria.

### 2.3 Randomization criteria

After confirming that the research subjects meet all the eligibility criteria and none of the exclusion criteria, the principal investigator or co-investigator will complete all the items described in the case registration form and send the registration form to the data center.

### 2.4 Study intervention

After registration, protocol treatment will be started within 28 days. Treatment will be performed for 2 days either with an auto-DODS followed by conventional-DODS or conventional-DODS followed by auto-DODS. The protocol treatment will be administered for 30 minutes at rest and for 6 minutes during a walk.

### 2.5 Standard procedures

The data center confirms the eligibility criteria and issues a “case registration confirmation,” which lists the result and the registration number. Furthermore, the data center will assign patients either to the “auto-DODS followed by conventional-DODS” or “conventional-DODS followed by auto-DODS” protocol according to a predetermined allocation list and will notify the principal investigator or co-investigator of the assignment.

### 2.6 Primary outcome measure

Primary endpoints are mean oxygen saturation (SpO_2_) (%) at rest and during the 6-minute walk test.

### 2.7 Secondary outcome measures

Secondary outcomes are described below.

(1) Percentage of time when auto-DODS operates at each sensitivity at rest and during the 6-minute walk test (%).
(2) Percentage of time when SpO_2_ falls below 90% at rest and during the 6-minute walk test (%).
(3) Times when SpO_2_ falls below 90% at rest and during the 6-minute walk test (minutes).
(4) Minimum value of SpO_2_ at rest and during the 6-minute walk test (%).
(5) Mean pulse rate at rest and during the 6-minute walk test (per minute).
(6) Maximum pulse rate at rest and during the 6-minute walk test (per minute).
(7) Six-minute walking distance (m).
(8) The time taken to recover SpO_2_ after 6 minutes of walking to the level obtained at rest (minutes).
(9) Borg scale.
(10) Comfort and reliability measured by a numerical rating scale and a questionnaire, respectively.

### 2.8 Participant timeline

The study period is from the initial publication date of the Japan Registry of Clinical Trials (jRCT) to May 31, 2021. The registration period is from the initial publication date of the jRCT to April 30, 2020. The follow-up period is 2 months from the date when the final case is registered. The date to publish the abstract of the summary report will be May 31, 2021.

### 2.9 Sample size

The sample size was determined as 26 cases (13 cases per group) in consideration of feasibility within the study period. The main purpose of this clinical trial is to clarify the efficacy of the auto-DODS equipped with a Hi-Sanso Portable α II for patients who need LTOT during the day. Since no prior study has been conducted on LTOT-requiring patients using this device, the number of subjects based on statistical tests was not determined. Tiep et al reported that the standard deviation of SpO_2_ in conventional-DODS on effort was 3.8% and higher than that in conventional-DODS at rest.^[1]^ Referring to this value, for 26 cases, the width of the 95% confidence interval of the difference between the mean SpO_2_ of the auto-DODS and of the conventional-DODS is 6.15%. Since this is a crossover trial, the 95% confidence intervals will be less than 6.15% for the difference in SpO_2_ at rest and on effort.

The period will not be extended even if the sample size is not fulfilled during the study period.

### 2.10 Allocation and sequence generation

In this trial, a permuted block method is used in which the block size is concealed by the biostatistician until the data analysis is performed. The person in charge of statistical analysis generates the allocation list. The data center receives the created allocation list and performs the allocation with concealment for the sequence. The person in charge of statistical analysis then stores the allocation list.

### 2.11 Blinding

This trial is an open-label trial.

### 2.12 Data collection and management

For any subjects who discontinue testing during the study period, data collected before the discontinuation will be used as research data. However, the data will not be used if consent has been withdrawn for use of the data from the participant. If there is any doubt about the eligibility of the data, the responsible biostatistician and the principal investigator will discuss the matter and decide on the next course of action.

### 2.13 Statistical methods

Analysis will be performed after the use of the test device has ended in all cases and after the data has been fixed.

### 2.14 Descriptive analysis of patient groups at baseline

Distribution and summary statistics of the background data of the study subjects will be calculated for the full analysis set, the per protocol set, and the safety analysis target population. For nominal variables, category frequencies and proportions will calculated for each group. For continuous variables, summary statistics (number, mean, standard deviation, minimum, median, and maximum) will calculated for each group.

### 2.15 Data analysis of the primary endpoints

In this study, a serious carryover effect will not be present in that we will use data from 30 minutes at rest and 6 minutes on effort, and a 4-week interval will be imposed after the first treatment period. The difference between the mean value of SpO_2_ of the auto-DODS and the conventional-DODS and its 95% confidence interval will be estimated as the primary analysis for the primary endpoint. This analysis will performed for the data at rest and on effort.

### 2.16 Analysis pertaining to the secondary evaluation criteria

To support the main analysis, the same analysis used for the primary endpoint will be conducted for the secondary endpoints. In addition, an exploratory statistical test will be performed for the primary endpoint and secondary endpoints, which will not be performed to confirm the research hypothesis because of the limited sample size. The statistical test will consist of a 2 sample *t* test for the difference in the observed values obtained from the 2 periods, which is valid under a model that assumes the observed values of device, period and subject with no carry-over effect. Furthermore, using the data of the first period, the difference between the average value of each endpoint of the auto-DODS and the conventional-DODS and their corresponding 95% confidence intervals will be estimated, and the *P* value will be calculated based on a *t* test or Wilcoxon test.

### 2.17 Data monitoring

Monitoring will be conducted regularly to confirm that the research is being conducted safely and in accordance with the research protocol and that data are being correctly collected. The principal investigator will appoint a person responsible for the monitoring of the tests. This person must have an educational history on regulatory requirements such as the “Clinical Research Law” and fully understand the contents of the research plan, consent documents and monitoring procedure. During the study period, the monitor will directly check source documents (consent sheet, medical record, case report, etc). The items to be confirmed by monitoring will be defined in the monitoring protocol. The monitor performs his/her duties before, during and after the trial (discontinuation / termination).

### 2.18 Harms

Even if subjects participate in this clinical study, the number of tests performed and blood samples collected will not be increased (aside from the 6-minute walk test) beyond what is expected from routine medical care. One of the drawbacks of participating in this trial is that when the device is stopped due to battery exhaustion, power failure, etc, oxygen will not be supplied for a long time, and symptoms associated with hypoxia and oxygen deficiency may occur. To minimize the risks and demerits of diseases suffered in this clinical trial, “inclusion criteria” and “exclusion criteria” are carefully considered. In addition to monitoring whether the disease that occurred is within expectations, if a serious or unexpected disease occurs, it will be carefully examined and reviewed, and necessary countermeasures will be taken. The clinical research insurance pays compensation for death or certain residual disability caused by this clinical trial. The cost of treatment given for other health hazards is generally paid by the patients’ health insurances or their own financial means.

### 2.19 Auditing

To guarantee the quality of the clinical trial, the auditor will evaluate whether the clinical trial is conducted in compliance with the research plan and procedure, independently of and separate from the normal monitoring and quality control operations. The principal investigator appoints the auditor to conduct the audit.

The principal investigator and institution will provide all clinical research-related records, such as source materials, for direct review during monitoring, audits, accredited clinical research review and regulatory review related to the clinical research.

## 3 Ethics and dissemination

### 3.1 Consent or assent

In this clinical trial, consent will only be obtained from the subjects of the study, not from the subject's legal representatives. The principal investigator will create a consent form as simple as possible. If there is any information that may affect the intention of the subject to continue with participation in the trial, the principal investigator will promptly revise the consent form according to the revised procedure manual. The principal investigator or co-investigator will give the candidate an explanation using the consent form, an opportunity for the candidate to ask questions and enough time to decide whether to consent. After confirming that the candidate has understood the contents of the clinical trial well, the principal investigator or co-investigator will obtain written consent. The principal investigator or co-investigator will obtain re-consent from the participant if the consent statement has a revision that may affect the participant's willingness to participate.

### 3.2 Patient and public involvement

Neither patients nor the public were involved in the design or conduct of the study.

### 3.3 Confidentiality

Data will be handled according to Japanese law. All original records will be archived at trial sites for 10 years. A clean database file will be anonymized and maintained for 10 years.

### 3.4 Dissemination policy

The protocol will be reported according to SPIRIT guidelines.^[3]^ The results of this clinical trial will be submitted as a paper in parallel with a presentation at a research conference. All co-authors will review the content of the article before submission, and only those who have agreed to the content of the presentation will be co-authors.

## 4 Discussion

Since conventional-DODS detects inhalation based on a predetermined sensitivity, any inhalations will not be detected if they fall below this sensitivity level. On the other hand, auto-DODS is expected to detect a wide range of inhalations by automatically switching among multiple sensitivities according to the situation. We focused on this difference and planned this trial based on the hypothesis that auto-DODS enables the detection of inhalation more accurately than conventional-DODS, and as a result, that auto-DODS improves oxygenation.

The limitation of this study is the relatively small sample size, which is not based on statistical testing because of a lack of reports about the efficacy of auto-DODS.

## Author contributions

**Conceptualization:** Kazuyuki Kobayashi, Takashi Omori, Takehiro Otoshi, Kanoko Umezawa, Naoko Katsurada, Masatsugu Yamamoto, Motoko Tachihara, Yoshihiro Nishimura.

**Data curation:** Tatsuya Nagano, Takehiro Otoshi, Kanoko Umezawa, Naoko Katsurada, Masatsugu Yamamoto.

**Formal analysis:** Tatsuya Nagano, Takashi Omori, Yoshihiro Nishimura.

**Funding acquisition:** Tatsuya Nagano and Yoshihiro Nishimura

**Investigation:** Tatsuya Nagano, Takehiro Otoshi, Yoshihiro Nishimura.

**Methodology:** Tatsuya Nagano, Takashi Omori, Yoshihiro Nishimura.

**Project administration:** Tatsuya Nagano, Yoshihiro Nishimura.

**Resources:** Tatsuya Nagano.

**Software:** Tatsuya Nagano.

**Supervision:** Tatsuya Nagano, Kazuyuki Kobayashi, Takashi Omori, Yoshihiro Nishimura.

**Validation:** Tatsuya Nagano, Motoko Tachihara.

**Visualization:** Tatsuya Nagano.

**Writing – original draft:** Tatsuya Nagano, Kazuyuki Kobayashi, Takashi Omori.

**Writing – review & editing:** Tatsuya Nagano, Kazuyuki Kobayashi, Takashi Omori, Takehiro Otoshi, Kanoko Umezawa, Naoko Katsurada, Masatsugu Yamamoto, Motoko Tachihara, Yoshihiro Nishimura.

All authors read and approved the final manuscript.
